# Supplementary material for: Disodium Fumarate Alleviates Endoplasmic Reticulum Stress, Mitochondrial Damage, and Oxidative Stress Induced by the High-Concentrate Diet in the Mammary Gland Tissue of Hu Sheep
Source: Antioxidants (Basel). 2023 Jan 18;12(2):223. doi: 10.3390/antiox12020223 (PMC9952365; doi:10.3390/antiox12020223)
Supplement: Supplementary file 1 [file antioxidants-12-00223-s001.zip › antioxidants-2144186-supplementary.pdf]

Supplementary Table S1 Primers used in quantitative real-time PCR analysis

Genes	Forward primer	Reverse primer	Size (bp)	Gene bank accession
AKT1	CCCTGGATTACCTGCACTCG	CTTGCACAGTCCAAAGTCGG	113	XM_027956972.2
CAT	AAGCTGGTTAATGCCAA	ATGGCATTGAAAAGATCTCG	146	XM_004016396.5
NQO1	CTTTCAGTATCCTGCCGAGA	TACTTGTAAGCGAACTCCC	192	XM_012190046.4
HMOX1	CATGCCCCAGGATTTGTCA	TTCTCCTTGTTGCGTTCGAT	189	XM_027967703.2
GPX1	GCTTCCCGTGCAACCAGT	TCGCCATTCACCTCGCACT	136	XM_004018462.5
GSS	ACATATTTGACCAGCGTGCC	GCCATCCCGGAAGTAAACCAC	161	XM_042230081.1
ATF4	AGACAACAGCACGGAGGATG	TCTGGCATGGTTTCCAGGTC	120	XM_012158819.3
ATF6	CGTCAGCGTTACGGAGTAGT	AACAGGGCAGAATCCCAGTC	179	XM_012185020.3
eIF-2α	GAAAGCTGCAAAGCAGGAAGC	AGCCTCCCACCTTCTTTAGG	155	XM_042235702.1
PERK	GCAGTGGCAATGAGAAGTGG	CCAATCTGCAACGGAGACCT	200	XM_004005901.5
GRP78	GGATCATCAATGAGCCGACAG	ACCCAGATGAGTGTCTCCGT	173	>XM_004005637.4
CHOP	ACCACACCTGAAAGCAGATCCT	GGTGCCCCGATTTTCATCTG	146	XM_027974337.2
IRE1α	ATGCTTGTTCGATTGCTGCT	CGTAAAGGGAAGTTTCGTCAGG	291	XM_027974337.2
GAPDH	GGGTCATCATCTCTGCACCT	GGTCATAAGTCCCTCCACGA	176	NM_001034034.2

Supplementary Table S2 Antibody information for Western blot determination

Name of antibody	Dilution Ratio	Article Number	Manufacturers
VDAC1	1:1000	66345-1-Ig	Proteintech
IP3R	1:1000	A4436	Abclonal
GRP75	1:500	A0558	Abclonal
MCU	1:500	AF16281	Abclonal
GRP78	1:1000	AF0171	Beyotime
CHOP	1:1000	AC532	Beyotime
PERK	1:1000	A21255	Abclonal
ATF4	1:1000	AF1048	Beyotime
ATF6	1:1000	AF6243	Beyotime
IRE1α	1:500	AI601	Beyotime
p-PERK	1:500	AF5103	Affnity
p-IRE1α	1:1000	AF8208	Beyotime
eIF-2α	1:500	AF7200	Beyotime
MFF	1:500	A8700	Abclonal
Drp1	1:500	A16661	Abclonal
Fis1	1:500	A5821	Abclonal
MFN2	1:500	A12771	Abclonal
MFN1	1:500	A9880	Abclonal
OPA1	1:500	A9833	Abclonal
SIRT1	1:1000	AF1267	Beyotime
PGC-1α	1:500	A12348	Abclonal
TFAM	1:500	A3173	Abclonal
NRF1	1:500	A3252	Abclonal
CAT	1:500	A11220	Abclonal
HO-1	1:500	AF1333	Beyotime
Nrf2	1:500	AF7623	Beyotime
phospho-Nrf2	1:1000	AF1609	Beyotime
GPX1	1:500	A11166	ABclonal
NQO1	1:500	A19586	ABclonal
Actin	1:1000	AA128	Beyotime
GAPDH	1:50000	60004-1-Ig	Proteintech
